# Selections

**Published:** 1817-06

**Authors:** 


### PART III.

### SELECTIONS.

—— Factorum est copia nobis.

### FOREIGN JOURNALS.

1. Pathology. *Observations recently made at Martinique and Guadaloupe, on the negroes and people of colour addicted to the habit of eating Earth.** It has been some time known, from the reports of missionaries, that there exists among the inhabitants of different countries of the torrid zone, the extraordinary habit of eating earth. And the attention of the scientific traveller has been strongly aroused to this subject by the interesting details which Baron Humboldt has given of the Ottomacs of the Orinoco, and by those of M. de Leschenault respecting the Javanese.

This singular depravation exists in all the islands of the western Archipelago. A long residence in Martinique and Gaudaloupe has offered to the notice of M. Moreau de Jonnès numerous examples of it. He considers it as independent of want of food, and produced by morbid causes, general and permanent.

The individuals, exhibiting it, are almost exclusively negroes or people of colour. It is rarely seen among the whites; and, whenever attacking them, it seems to be the effect of changes in the animal economy resulting from previous diseases. Pregnancy and amenorrhœa sometimes produce it in females of this cast. But there is no doubt that the disturbance, thus excited in the female constitution, is the cause of it; since, in Europe, analogous propensities arise under similar circumstances.

In individuals, of African origin, the habit of eating earth appears not to be, as in whites, the effect, but the immediate cause, of disease.

This difference, doubtless, results from that of their respective modes of diet. Succulent food, and especially the employment of spirituous liquors, probably go far to prevent, in whites, the gastric affections from which this strange appetite results; nor are they exposed to it except when a state of distress, rarely seen in these islands, compels them to change their wonted diet.

---

* Observations faites récemment a la Martinique et la Gaudeloupe, sur les nègres et gens de couleur adonnés a l'habitude de manger de la terre. Par l'aide-de-camp Moreau de Jonnès, &c. *Bulletin de la Société Medicale d'Emulation.* *No. V.* *Mai* 1816.

In the natives of Africa, the use of dried fish and of food almost exclusively vegetable, seems to favour the physiological disposition produced by the climate. Hence, wherever the singular propensity of eating earth has been observed, those who have contracted it, will be found living on a diet from which animal substances and spirituous liquors are almost utterly excluded.

Connecting this observation with that of the site of countries inhabited by people addicted to this propensity, and which are all placed under the torrid zone, we may conclude that the vital forces, unceasingly attracted from the centre to the circumference by the excitement of an ardent temperature, leave the visceral system in a sort of atony, from whence result the changes which probably give rise to this depraved appetite.

This is a conjecture the truth of which can only be settled by the observation of the enlightened physician; but, however it be determined, we may, at least, trace with precision the effects of an unknown cause.

The morbid dispositions, which probably precede the desire of eating earth, have eluded the observation of the French writer. Great attention and acuteness would probably be necessary to detect them. Yet many facts authorize the supposition that this habit is particularly developed in individuals, of decidedly lymphatic constitution and lax fibre, possessing a frame imperfectly *animalized*, and mind and faculties generally inert. In those, who had not long contracted this mania, no gastric affection was detected on careful examination; but, in many others, who had been long subject to it, were combined the characters of visceral and intestinal diseases: their skin was parched, of an earthy aspect, and of a yellow tinge more or less conjoined with the natural colour of the dermoid structure: their look was languid; respiration difficult; breath often infectious; extremities emaciated; pulse intermittent; abdomen swollen, and at times painful. The general aspect of the body was that of suffering. Indolence, and difficulty of every kind of motion, were also strongly marked.

The unfortunate beings, thus circumstanced, still retain the insatiable desire of indulging the mania, which, they are aware, serves only to aggravate their sufferings; and, when the habit becomes confirmed, their thoughts are only occupied with the means of eluding the vigilance exercised towards them.

These precautions often compel them to satisfy their appetite with the first earth within their reach; of which they devour great quantities Yet it is certain that they act thus rather from necessity than choice, or from a want of discernment or knowledge, the more frequently exhibited by younger subjects, as, this noxious propensity being considered criminal, its details are enveloped in a sort of mystery.

Although the writer has seen pieces of carbonate of lime, already in part eaten, seized upon a girl, of twelve years; yet reiterated observation has convinced him that the individuals, attacked by this mania, do not swallow every species of earth indiscriminately.

That which constitutes the particular object of their taste in the islands of Martinique and Guadaloupe, is an earth, composed of argil, silex, and magnesia, in little variable proportions, and more or less coloured with oxide of iron. Its specific characters are determined by the relative quantities of its elements. In general, it bites the tongue, reddens in the fire, exhales a smell of alumina, readily petrifies with water, splits on desiccation, appears unctuous to the eye and touch like steatite, and presents, according to the proportion of its ferrugineous parts, different shades of ochry red.

This earth proceeds from the decomposition of the porphyroid lava thrown up by the ancient volcanos of the Antilles, in currents, the length of which is sometimes five or six thousand toises; and their elevation, two or three hundred.

Argil constitutes the basis of this lava: it contains, moreover, pyroxene, amphibole, hexagonal mica, and coarse felspar, which forms the greatest part of its mass.

The magnesian earth, contained in the mica, is liberated by the decomposition of the lava, and forms, by its mixture with argil, a steatitic earth, which is soapy and fat, particularly when moistened. These characters have not escaped the inhabitants of the islands. They call every projection, the surface of which is formed by this earth, *morne-savon*; and the name signalizes to travellers, particularly in the rainy season, roads, the rapid descent of which is slippery and dangerous.

The character of unctuosity, which distinguishes this species of argil, doubtless, diminishes its arid and earthy taste; although this difference is far more perceptible to the touch than to the tongue.

The unctuosity, which seems, in some degree, to connect this earth with animal and vegetable substances, has, perhaps, contributed to lessen the natural repugnance to feed upon a substance utterly destitute of all alimentary properties.

It is remarkable, that this earth resembles, in some degree, if not perfectly, the *terra sigillata* of Lemnos, so celebrated in antiquity. It is, like that, of volcanic origin; and probably would not exercise more pernicious effects upon the animal œconomy, if it were not eaten by those of the western islands, who use it in considerable quantities. The stomach, once habituated to this load, can no longer bear its privation without experiencing painful contractions, which re-excite the appetite for it. The frequent, or almost continual presence of an absorbent earth in the intestinal canal, exhausts the gastric fluid; at first impairs, and soon subverts, the digestive process; and induces, under a burning climate, dysenteric maladies, always incurable and speedily destructive.

The medicinal use of the sigillated boles proves, that mischief only arises from the abuse or unduly prolonged employment of this earth: and the consumption of it by the natives of Africa not being attributable to want of food in islands abounding with the means of human subsistence, this appetite may, perhaps, be regarded as a sort of natural instinct, which incites to the use of an absorbent substance, men suffering from all the disadvantages of a pituitous temperament, developed by a humid climate.

From this consideration, it may be inferred, that a propensity, so inevitable and destructive, might be advantageously combated, by introducing, among other regulations which policy and humanity indicate, into the diet of the negro slaves of the western colonies, the habitual use of a diluted spirituous liquor. Rum, as being procured on the spot, here offers an excellent and very cheap resource. Many planters have already begun to distribute among their negroes, as a daily ration, a certain quantity of it, which is mixed with water in their presence.

This practice might, perhaps, be advantageously adopted, especially in marshy countries, as in Guiana and some parts of Martinique and Guadaloupe. Its general establishment is much to be desired. In individuals of lymphatic constitution, it would serve to recall periodically towards the epigastrium the vital forces, rendered incessantly divergent by the exciting influence of the climate on the cutaneous surface.

Assisted by the execution of the different measures, which have occupied the attention of eminent statesmen, this plan would, doubtless, obviate a perversion of appetite, which adds yearly to the ravages sustained by a valuable part of the population of our colonies.

---

*Additional Note by Messrs. Breschet and Cloquet.*—The singular disease, exhibited in the preceding Memoir, appears to be much more frequent in equatorial than northern regions. This, perhaps, depends on the want of real aliment being much less strongly felt under the torrid zone than in cold or temperate climates. Yet they think that the act of earth-eating is not decided by a peculiar taste, but by imperious necessity. Many nations, widely remote from each other, have the habit of allaying hunger by the ingestion of pure earth. M. de la Billardière asserts, that the inhabitants of New Caledonia possess only this kind of food during certain seasons of scarcity. When turtle-fishing is prevented by the inundation of the Orinoco, which lasts about three months, the Ottomac nation is reduced to feed almost exclusively on a species of clay. Humboldt, by whom this fact is stated, asserts, that about a pound and a half of it, unmixed with crocodile fat, or any vegetable substance, is daily consumed by each individual. The only preparation consists in slightly frying, and afterwards moistening it.

Somewhat analogous is observed by Golberry respecting the negres of *Los Idolos*, islands at the mouth of the Senegal. They mix with their rice a mineral substance, which seems to serve as a substitute for butter.

Brown says, that the crocodiles of South America swallow both small stones and pieces of wood, when the lakes which they commonly inhabit are dry, and they want food.

Not far from Krasnoiarsk, on the river Jenissey, and in some mountains bordering on the Amour, is found a substance, called by the Russians, *kamennoïé maslo*, or rock butter. The elks and goats are singularly fond of it, and Patrin reports that it is employed by the hunters, as a bait wherewith to catch these animals.

From all these facts may be drawn a somewhat curious general conclusion:—The earths or stones, employed to distend the stomach, to allay hunger, or satisfy a depraved appetite, are almost invariably unctuous to the touch, fat, homogeneous, and contain much magnesia or alumine. Thus the author of the memoir has discovered that, at Martinique and Guadaloupe, the substance devoured by the negroes was an earth analogous to steatite, and formed by the decomposition of the porphyroid lava of the old volcanos of these islands.

Vauquelin has analysed that of New Caledonia, and found it to consist of 0,37 of magnesia; 0,36 silex, and 0,17 oxide of iron. It is a green steatite, friable and tender.

The earth of *Los Idolos*, is also a real steatite; but white, soft and unctuous. Golberry ate of it without inconvenience or disgust.

The rock butter forms stalactites in the cavities of the mountains alluded to. It is a mixture of argil, sulphate of alumine, sulphate of iron, and a small quantity of petroleum.

One of these gentlemen asserts, that once, when pressed by hunger, he ate about five ounces of a lamellated talc, of a silver green colour, and very flexible, abounding in the mountains of the Tyrol. His appetite was satisfied without the slightest inconvenience.

Most of the varieties of bolar and sigillated earths, which have been so long and frequently vaunted in the treatment of internal diseases, belong to the same class; but it is very probable that their medical virtues depend upon the iron which enters into their composition.

---

### *Enlargement of the Brain, exhibiting all the Symptoms of Hydrocephalus.**

Henrietta Zollner, aged about five years, displayed a somewhat singular physiognomy. The cranium was disproportionately large,

* *Vergrösserung des Gehims, die alle Zufälle der Gehimwassersuchte darstellte, behandelt und aufgeseichnet*, von Dr. Teller. *Journal der Practischen Heilkunde.* November, 1815. The design of inserting a translation of this case in the Selection Department of our Journal, was announced in October last, by one of our Editors (See *Dr. Palmer's Case of diseased Brain*, vol. ii. p. 278). Though prepared at that time, it has hitherto, from some accidental circumstance, been omitted. The appearance of it in a contemporary Journal has served to recall our attention to an almost forgotten promise; and we here insert the case, which will, and the forehead projected greatly. She possessed a weakly constitution, but had always been free from illness, except that, nine months previously, she had suffered from chincough; of which, however, she had been, for some weeks, perfectly cured at the institution (with which Dr. Teller was connected).

On Sunday, June 21st, she began to complain; but continued to go about even on the ensuing day. The whole of Tuesday she kept her room; and whether still or in motion, constantly reclined her head. She was, moreover, peevish, and continually drowsy. During the night she was extremely restless, and raised her hand, particularly the left, frequently to the head. Even during health, her sleep had been much disturbed.

On Thursday the 25th, vomiting and diarrhœa came on. The intestinal discharge was greenish and very offensive, that from the stomach greenish and slimy. An emetic was administered by the surgeon: it operated once; after which the vomiting ceased, but the diarrhœa still continued.

On Monday the 29th, her parents brought her to the Institution: of the exciting cause of the symptoms they could afford no information. The girl was very feverish, had great heat, and a rapid pulse. Under the suspicion that the disease was dropsy of the ventricles of the brain, probably complicated with worms, a mixture was prescribed, composed of a decoction of *lovage* and *worm-seed*, with *citrate of potash;* and a powder of *zinc*, *digitalis*, *henbane*, and *liquorice*.

On the 30th, the girl came under the care of Dr. Teller. He found her drowsy. On being roused, she beat about with her hands, returned no answer to her mother's questions, and groaned almost incessantly. The pulse was full, not too rapid, about 90 in a minute. The forehead was uncommonly hot, and respiration somewhat embarrassed. From inquiry respecting the symptoms which precede dropsy of the brain, it could only be learnt that the child was uncommonly keen for her age; but she had never been observed to stumble in her gait. The medicines, before prescribed, were continued; and four leeches were applied behind the ears. About four o'clock in the afternoon, the child was much worse: her pulse was small, 125 in a minute; her countenance of a yellowish pale hue; and her lethargy such that it was no longer possible to arouse her. The leeches had drawn well, and the bleeding still continued very profuse. They had been applied about noon. On that account, the application of a sponge, moistened with vinegar, was immediately directed. A blister to the neck, and lotions of cold water to the head, were also prescribed. At five

---

perhaps, be read with interest, not only from its peculiarity, but from the specimen it affords of the practice of German physicians. Dr. Hufeland, in a note appended to the paper, promises, at some future time, "to enter more at large upon this disproportionate increase of the brain, a morbid condition hitherto not regarded; which affects all the symptoms of hydrocephalus, and is even, in my opinion, capable of producing them." ED.

o'clock, the child was in an agony. The bleeding had not been completely suppressed by the application of the vinegar. Decoction of *lovage* with *valerian*, and a powder with calomel, opium, and musk, were now prescribed. For one whole hour, the cold lotions were persevered in without effect. Therefore, the *spiritus aromat. camphorat* Ph. p. was directed as a wash to the whole body, and lotions to the face. About seven o'clock, the child exhibited some distortion of the facial muscles, and died.

Dissection on the 1st. of July.

1. *Examination of the Head.* The brain was uncommonly large for the age of the child, but not loaded with blood. The ventricles possessed their natural capacity, and contained no fluid. The plexus choroides were rather destitute of blood than gorged. Every thing else was in a natural condition.

2. *The Thorax* The lungs were quite sound, very pale, and deficient in blood. A small portion of the left lung was of its naturally dark colour, and contained somewhat more blood. The heart was in its usual state but bloodless. From the vena cava itself little blood was discharged on incision.

3. *The Abdomen.* All the viscera were sound, but unusually pale and bloodless; except the spleen, which appeared natural. The stomach was very large for this age. No other vestige of disease was observable; except that some of the mesenteric glands were considerably enlarged; yet without any appearance of hardness or suppuration.

### FRENCH DICTIONARY of MEDICAL SCIENCES.

### II. Obstetrics. *Successful Case of Puncture of the Uterus**

A woman, aged 23, of lymphatic temperament, and who, some years before, had been fortunately delivered of one child, experienced anew all the symptoms of pregnancy. Six weeks after their appearance, in consequence of some rough treatment sustained in a frolic, she was distressed with a vaginal discharge, pains in the renal and inguinal regions, sensation of weight in the perinæum, and with difficulty of walking and voiding the fæcal matter. M. Jourel, consulted in about a fortnight, recommended rest, and the use of slightly astringent liquids. The woman went into the country, and he did not see her again for a month.

At the end of this period, she informed him that the hæmorrhage had only ceased within the last two days; but that all the other symptoms had progressively increased so as to render evacu-

* *Dictionaire des Sciences Médicales.* Tome, ix. *Article, Deviation.* Puncture of the uterus has always, from its first suggestion by Dr. Hunter, been regarded as a very formidable operation, and never, we believe, been attempted by any Bristish practitioner. M. Jourel, of Rouen, has performed it in France with equal intrepidity and success. Some notice of it will probably be acceptable to the readers of this Journal. Edit.

ation of the urine and fæces very difficult. An attempt was, therefore, made to relieve her by catheter and injection. Six days after her return, September 18th, 181[illegible], on the introduction of a finger into the vagina, was discovered *a firm tense body, possessing the figure of the uterus in the earlier stages of gestation; the large extremity of which compressed the rectum; and the smaller, the bladder against the posterior surface of the pubes.* By these signs, M. Jourel and one of his colleagues recognized a retroversion of the uterus. Having evacuated the contents of the bladder and rectum, they endeavoured by the introduction of three fingers, first into the vagina and then into the intestine, to replace the deranged organ. *Low diet* and *tepid baths* were prescribed.

Next morning, assisted by two other gentlemen, they placed the woman, on coming from the warm bath, upon her elbows and knees, and renewed their useless efforts at reduction with the whole hand introduced into the rectum. *Venæsection and tepid baths.* The same evening, a fruitless attempt was made to pass a catheter by the uterine orifice, in order to burst the membranes and discharge the liquor amnii: the curvature of the neck of the organ presented an insuperable obstacle to this unprecedented manœuvre. Under these embarrassing circumstances, they preferred to Gardien's operation of pubic synchondrotomy, that of puncturing the uterus through the posterior boundary of the vagina, as proposed by Dr. Hunter and other succeeding writers, but hitherto unattempted.

M. Jourel operated with the common trocar passed along the fore-finger of the left hand. About one pound of bloody fluid issued from the canula: a quantity greater than is commonly said to exist at this period of pregnancy; but which the peculiar circumstances of the retroversion sufficiently explain: *ubi stimulus, ibi fluxus.* The uterus immediately became softer, the pulse less frequent; and the general condition of the woman improved. *Anodyne draught, tepid bath, and emollient injection.* Reduction was not immediately attempted, the woman being much fatigued.

From this time, the urine was freely evacuated and sleep returned. On the morrow, things continued in the same state. Much serum oozed from the vagina. The uterus becoming somewhat painful, *narcotic injections and emollient fomentations were* employed.

The pulse, on the 17th, was more small and frequent; the face pale; the hypogastric region painful to the touch; the uterus more hard and tender. The difficulty of voiding urine returned in the forenoon; and vomiting twice came on, accompanied with extrication of intestinal flatus. The strength sank. The discharge was suppressed, but re-appeared on the ensuing day. The stools were liquid. Towards evening, the symptoms were mitigated, and continued to subside, with the exception of great debility The emission of urine was partly involuntary.

Typhoid symptoms became evident on the 22d, with a greyish and putrid discharge from the vagina, and a copious and involuntary flow of urine in the vertical position. *Glysters of cinchona and tonic injections.* By the help of tonics, the strength recruited on the subsequent days. The fœtid discharge occurred only at intervals. This, in addition to the others, was a certain sign that the bladder had not been punctured instead of the uterine organ. It was not until the 27th that the neck of the uterus resumed its natural situation in the middle of the pelvis. On the 2d of October, the organ returned to its pristine volume. Its orifice was directed towards the sacrum, and the vaginal discharge greatly diminished. A white fluid, resembling the pus of phlegmon, was voided by the rectum. The pulse was accelerated. Towards evening the patient had a slight paroxysm of hectic fever. The puriform matter enveloped the excrements, and issued profusely before their expulsion. This symptom gradually diminished, and ceased altogether on the 10th of the same month.—The patient then went to the country for three weeks; during that interval, and till the 15th of December, when menstruation was restored, she experienced a distressing tension of the abdomen with transient fits of colic. The stools differed greatly in consistence. After the re-establishment of the menstrual flux she enjoyed good health.

This case proves, (say the Professors Dubois and Désormaux, appointed to make report of it) that even under those unfortunate circumstances, where the surgeon is summoned too late to employ with success the milder remedies, such as bleeding, baths, emollient fomentations, and evacuation of the bladder and rectum, the puncture of the uterus may be practised with some prospect of recovery.

It is also seen that, at the period of pregnancy, when this operation was executed, the remains of a fœtus may be dissolved and discharged with the fluids secreted by the membranes, without their being distinctly perceived.

---

### Medical Jurisprudence.—*Article, Blessure.*

( Concluded from page 344. )

II. *The species of lesion with respect to the parts injured.*—In determining the degree of fatality of a lesion, it is necessary to consider both its situation and mode of infliction, with other circumstances which may exercise an influence upon it.

The French writer then proceeds to notice *lesions of the head*, both external and internal, and as inflicted by various instruments. From the importance of the organ, the peculiarities of its structure, and the obscure and equivocal character of the symptoms frequently connected with its derangement, great difficulties attend the investigation of these lesions. We need not enumerate their varieties. The consequences of them, as vertigo, head-ach, paralysis, epilepsy, defect or loss of intellect, may commonly be considered as incurable.

*Lesions of other parts of the nervous system* are dangerous in proportion to their vicinity to the brain. Complete division of important nerves, as the eighth pair, intercostal, and phrenic, are fatal; incomplete division dangerous. Wounds of all parts, liberally supplied with nerves, are often fatal from the irritation which they produce.

*Lesions of the sensual organs situated in the head* are rarely mortal. Wounds, however, near the supraciliary ridge, although slight, may induce blindness, from the pressure exercised by the cicatrix on the frontal branch of the fifth nerve.

*Lesions of the throat* are dangerous, according to the number and importance of the parts injured. Ligatures may be applied to all the vessels except the vertebral arteries; injuries of which are also invariably complicated with spinal lesions. Of lesions of the nerves we have already spoken: those of the laryngeal nerves sometimes induce aphonia.

*Lesions of the trachea* are not very dangerous, except when inflicted by fire-arms. Instances of recovery have been known, when the tube was completely divided. Those of the *œsophagus* are dangerous in proportion as they are lower down, and deeply implicate the canal and its appendages.

*Lesions of the thorax* differ in danger according as they are superficial or penetrating. Those of the osseous structure may be fatal by their effects on respiration, or by inducing pulmonary inflammation. Hæmorrhage and introduction of the external air into the thorax may be also the consequences of penetrating wounds. Contusions may induce caries of the bones, rupture of the lungs, heart, and large vessels, or inflammation terminating in pulmonary suppuration, sphacelus or effusion. The principal danger of *lesions of the pericardium* arises from the liability of that membrane to inflammation and effusion.

*Lesions of the heart*, not implicating the coronary vessels or cavities of the organ, though dangerous, are not invariably fatal; but of wounds of these, as of the nerves, death is the inevitable, yet not always direct, consequence.

*Lesions of the thoracic duct* are necessarily fatal; and those of the *thoracic portion of the œsophagus*, and those of the *diaphragm*, extremely dangerous.

*Lesions of the abdomen.* Those of the integuments are formidable from their implicating, or otherwise affecting the internal organs, from the danger to which the epigastric artery is exposed, and from the risk of inducing hernia, or tendinous inflammation and its consequences. Those of the internal parts are formidable from their impairing the digestive process, and producing various incurable kinds of effusion, and from affecting structures which exquisitely sympathize with the nervous system.

*Lesions of the omentum and mesentery* are only dangerous when implicating the large vessels, or when the injured organ, lodged in the wound, inflames and suppurates there.

*Lesions of the stomach* are not always fatal, but highly formidable from the hæmorrhage, inflammation, and nervous commotion, to which they may give rise. The system is unable to support long the disturbance or suspension of the gastric functions. The danger is always greater in proportion to the size of the wound, its propinquity to the gastric orifices, the fulness of the organ at the moment of its infliction, and the degree of commotion. Death may result, in this case, from the shock which paralyses the nervous system, from hæmorrhage, from inflammation and gangrene, or from effusion of the contents of the organ into the abdominal cavity. It is especially in wounds of the stomach that the opinion of the juridical physician must be decided by individual circumstances.

*Lesions of the intestines* are not always fatal, even when the tube has been divided with loss of substance; and may sometimes be cured without leaving an artificial anus, the inconvenience and dangers of which are always greater in proportion as it is situated nearer the origin of the canal. When small, they may adhere to the external wound. They are most dangerous when complicated with contusions, and particularly those of fire arms. The consequences of inflammation of a portion of intestine, strangulated in an exterior wound, are to be dreaded. The danger of *lesions of the liver and spleen* depends on the depth of the wound, on the hæmorrhage, inflammation, and possibility of giving vent to any collection of pus. These organs are sometimes ruptured by external violence, without the slightest trace of injury being perceptible on the surface. Death, more or less prompt, is always certain in these cases, when the large vessels are implicated. Such accidents, when the external violence has not been severe, commonly indicate a morbid defect of consistence in these organs.

*Lesions of the gall-bladder and ducts* are generally complicated with other severe injuries. Danger may arise from effusion of bile into the abdominal cavity, and its exclusion from the intestinal canal. In this view, wounds of the hepatic and common ducts are more dangerous than those of the cystic duct and gall-bladder; as, in the latter case, the effusion of bile into the abdomen may sometimes be prevented.

*Lesions of the pancreas* are formidable from the consequent escape of blood into the abdomen, and from the loss of its fluid; those of the *abdominal portion of the thoracic duct* obviously fatal.—Among the *lesions of the urinary organs*, those of the *kidnies* are, from their situation, the least dangerous; but, they are necessarily fatal when the large vessels are implicated.

*Lesions of the ureters*, when penetrating, cause incurable effusion of urine into the abdomen, succeeded by inflammation and sphacelus, and are necessarily fatal. All distinctions between wounds of the fundus and neck of the bladder, and between those of its fibrous and membranous structure, are futile. The thing is to ascertain whether any large artery have been injured; whether or not the situation of the wound permit the evacuation of blood or urine effused into the pelvis, or between the neighbouring muscles; whether there have been contusion, which is commonly followed by inflammation and sphacelus. Rupture of the distended bladder by external violence is necessarily fatal.

As to *lesions of the male organs of generation*, wounds of the spermatic vessels *in the abdomen* cause an incurable hæmorrhage; but wounds of them in other situations are only accidentally fatal. Contusions of the testicle may produce dangerous nervine or inflammatory symptoms. Wounds of the vesiculæ seminales, by obliterating the excretory ducts, may destroy the procreative faculty. Among the *lesions of the female organs*, those of the uterus, though not necessarily fatal, are formidable. Injuries of the impregnated organ frequently destroy both child and mother; the latter commonly by hæmorrhage, unless the prompt expulsion of the ovum permit the uterus to contract. Different lesions induce retroversion, prolapsus, and rupture, which may terminate in abortion, or fatal inflammation of the uterus.

*Fracture of the pelvic bones* is dangerous, from the collection of blood or other fluids in this region, and from the commotions of the spine and brain with which it may be complicated.

*Lesions of extremities*, as affecting organs not essential to life, were, at one time, considered not fatal; but the principle and the assertion deduced from it, are too vague and general to be indiscriminately adopted. The total or partial loss of a limb, not being under favourable circumstances fatal, belongs to the class of *lesions curable with derangement of function.* In determining the consequences of such derangement, the profession and mode of life of the cripple should be considered. *Lesions of the large vessels* are more dangerous, as they are nearer the centre of circulation; but, in the present state of surgery, they are generally curable, unless some peculiar circumstance exists, rendering an operation impracticable. Considerable lesions, and contusions of the *articular cavities* and of the *tendons* and *nerves*, are dangerous, from liability to the invasion of nervous symptoms and sphacelus. *Luxations and fractures*, with or without wounds and contusions, are less formidable, in proportion to their simplicity.

III. *The organic condition of the patient, and external circumstances.* These demand great attention, as they may have a powerful influence on the fate of an accused person. The *age* of the subject is, by no means, a point of indifference in judging of any lesion. The great excitability of youth induces a correspondent high susceptibility of impression, compared to that of more advanced age; and the abundance of blood, and activity of circulation, expose youth to more violent hæmorrhages, but renders it better able to sustain the loss. During this period, in fact, lesions which would prove fatal or imperfectly curable in older subjects, frequently admit of complete recovery. *Sex* merits consideration, not so much in respect to absolute as to relative sexual distinction. The excitability and abundance of humours are greater in the female than the male. All lesions, inflicted during pregnancy, are more dangerous than at other periods, not only from their injurious effect on gestation, but because this state generally retards the cure of wounds. *Temperament*, as it indicates different degrees of excitability, demands also a serious regard. *Habit and idiosyncrasy* cause lesions to affect some persons more violently than others, as is frequently observed in military punishments. Peculiarity of constitution may also oppose the employment or efficacy of a medicine, otherwise imperiously indicated. *Morbid dispositions, or diseases themselves*, often powerfully contribute to the fatal issue of a lesion. Thus wounds of the head are particularly dangerous in persons disposed to apoplexy; those of the chest, in subjects of phthisical constitution. All injuries are more severe when inflicted on an individual suffering from fever, syphilis, scrofula, or other morbid affection. *The condition of the patient at the moment of receiving the wound* ought not to be neglected. It may be of importance, for example, to determine whether he had fasted or recently taken food; whether he were inebriated, enraged, awake, or sleeping; whether in the erect or sitting posture. Lastly, as to *external circumstances*, the climate, season, state of the atmosphere, epidemic constitution, residence of the patient during his treatment, mode of life and regimen, are so many points, the influence of which should be rigorously weighed by the juridical physician ere he proceed to deliver an opinion.

*Welsch*, 8vo. Leipsic, 1660, and Nuremburgh, 1719; *Meibomus*, 4to. Helmstadt, 1674; *Bohn*, 8vo. Leipsic, 1689, and Amsterdam, 1710; *Ammanus*, 8vo. Frankfort, 1690, and Leipsic, 1701; *Stahl*, 4to. Halle, 1698; *Hoogvliet*, 8vo. Amsterdam, 1732; *Eisenbach*, 4to. Rostock, 1746; *Hebenstreit*, 4to. Leipsic, 1755; and *Platner*, 4to. Leipsic, 1784 and 1800; are the authors of the principal monographs on the judicial examination of wounds. The writings of *Foderé*, *Mahon*, and *Male*, on this subject, may also be consulted with advantage. Ed.

---

In giving an account of the articles in the French Dictionary, which have Medical Jurisprudence, or rather Juridical Medicine, for their subject, our labours have, at times, almost inconsciously assumed the character more of original discussion than of simple analysis. This has particularly been the case in the articles *Cadavre* and *Avortement* (see *Medico-Chirurg. Journal*, vol. ii. pages 68—146, 420—497): and some of our Correspondents, whose opinions on subjects of this nature we highly value, have, while expressing their decided preference for these articles, recommended that all the remainder of the series should be written on the same principle. In complying with this request, it will be necessary to transfer our future discussions on Juridical Medicine to the *Original Department*; where an article on this subject will appear whenever the pressure of more valuable matter from our Correspondents does not preclude its introduction. Innovation, for innovation's sake, we shall steadily, at all times, reject; but, when suggested by liberal and enlightened men, and obviously leading to improvement, no selfish feeling, or dread of unworthy imputation, will deter us from adopting it. Editors.

